# Reducing shade avoidance can improve Arabidopsis canopy performance against competitors

**DOI:** 10.1111/pce.13905

**Published:** 2020-10-19

**Authors:** Chrysoula K. Pantazopoulou, Franca J. Bongers, Ronald Pierik

**Affiliations:** ^1^ Plant Ecophysiology, Dept. of Biology Utrecht University Utrecht The Netherlands; ^2^ State Key Laboratory of Vegetation and Environmental Change Institute of Botany, Chinese Academy of Sciences Beijing China

**Keywords:** *Arabidopsis thaliana*, canopy architecture, competition, hyponasty, planting pattern, shade avoidance

## Abstract

Plants that grow in high density communities activate shade avoidance responses to consolidate light capture by individuals. Although this is an evolutionary successful strategy, it may not enhance performance of the community as a whole. Resources are invested in shade responses at the expense of other organs and light penetration through the canopy is increased, allowing invading competitors to grow better. Here we investigate if suppression of shade avoidance responses would enhance group performance of a monoculture community that is invaded by a competitor. Using different *Arabidopsis* genotypes, we show that suppression of shade‐induced upward leaf movement in the *pif7* mutant increases the *pif7* communal performance against invaders as compared to a wild‐type canopy. The invaders were more severely suppressed and the community grew larger as compared to wild type. Using computational modelling, we show that leaf angle variations indeed strongly affect light penetration and growth of competitors that invade the canopy. Our data thus show that modifying specific shade avoidance aspects can improve plant community performance. These insights may help to suppress weeds in crop stands.

## Abstract 2

Hyponastic response in canopies facilitates light interception, but also light penetration. Inhibition of this response to neighbours increased canopy biomass, canopy closure and suppression of competitors.

## 1 INTRODUCTION

Competition shapes many ecosystems and is also major factor in crop production systems in relation to weeds. Through the course of evolution, natural selection has typically favoured strategies that enhance individual plant fitness. One very well‐established example is shade avoidance. When plants grow close together at high planting densities, individual plants consolidate light capture by growing away from the shade cast by neighbouring plants: the shade avoidance syndrome (SAS) (de Wit, Galvão, & Fankhauser, 2016). Here we will study if loss of the adaptive shade avoidance traits at high density, can benefit group performance against invading competitors.

Nearby plants are first detected through the Red (R): Far‐red (FR) light ratio (R:FR) in the light reflected between plants, a ratio that decreases because of selective absorption of R light for photosynthesis and reflection of FR light 1990. Shade avoidance responses are typically elicited upon detection of a reduced R:FR ratio, and are further promoted by depletion of blue light when the canopy closes (Ballaré, 1999; de Wit et al., 2016). Shade avoidance responses include upward leaf movement (hyponasty), elongation of stems and petioles and inhibition of branching and tillering (de Wit, Keuskamp, et al., 2016 *;* Franklin, 2008 *;* Pierik & de Wit, 2013). Shade avoidance responses occur in most crop and wild plant species, including the genetic model plant *Arabidopsis thaliana* (Ballaré, 1999; Casal, 2012; Franklin, 2008; Gommers, Visser, St Onge, Voesenek, & Pierik, 2013; Martínez‐García et al., 2010). In response to low R:FR, phytochrome photoreceptors are inactivated (Ballaré, 1999; Franklin, Davis, Stoddart, Vierstra, & Whitelam, 2003; Kozuka et al., 2010) and this relieves their repression of Phytochrome Interacting Factors (PIFs) (Jeong & Choi, 2013; Leivar & Monte, 2014; Li et al., 2012), a class of transcription factors that promote the expression of growth promoting genes (Oh, Zhu, & Wang, 2012; Zhang et al., 2013). PIF4, PIF5 and PIF7 are the dominant PIF proteins involved in shade avoidance in Arabidopsis (Hornitschek et al., 2012; Li et al., 2012; Lorrain, Allen, Duek, Whitelam, & Fankhauser, 2008; Pantazopoulou et al., 2017). Upon their activation in shade, PIFs readily promote a downstream pathway leading to increased elongation growth, primarily through the plant hormone auxin. Although PIF4, PIF5 and PIF7 can all bind promoter regions of auxin‐associated genes, PIF7 is especially important for induction of auxin biosynthesis enzyme‐encoding YUCCA genes. This in turn stimulates synthesis of auxin, which is then transported from the leaves to the petioles and hypocotyl to promote low R:FR‐induced elongation and hyponasty (de Wit, Ljung, & Fankhauser, 2015; Michaud, Fiorucci, Xenarios, & Fankhauser, 2017; Nozue et al., 2015; Pantazopoulou et al., 2017; Won et al., 2011). More specifically, PIF4 and PIF5 promote auxin responsiveness upon low R:FR while together PIF4, PIF5 and PIF7 regulate auxin production to respond to low R:FR (de Wit, Keuskamp, et al., 2016; Hornitschek et al., 2012; Li et al., 2012; Michaud et al., 2017; Pantazopoulou et al., 2017). Studies using mutants of these three transcription factors under low R:FR showed that, *pif7* mutants do not respond to low R:FR with hypocotyl elongation or hyponasty, whereas *pif4 pif5* double mutants have a reduced but not absent response (Hornitschek et al., 2012; Li et al., 2012; Pantazopoulou et al., 2017).

Shade avoidance responses are adaptive in dense vegetation (Schmitt, 1997; Schmitt, Stinchcombe, Heschel, & Huber, 2003), explaining how these responses have emerged so commonly in most land plants. They increase light capture of those individuals that express the shade avoidance traits best, thus enhancing light capture, growth and reproduction in the successful individuals (Dudley & Schmitt, 1996; Schmitt, Dudley, & Pigliucci, 1999; Schmitt, McCormac, & Smith, 1995). Plants that do not express these responses, or express them weaker than their direct neighbours will be suppressed and have severely reduced fitness. In specific environments, such as the forest understory, shade avoidance responses may not be adaptive since the severe growth investment does not yield a benefit; the tall foliage cannot be escaped from (Gommers et al., 2013; Valladares & Niinemets, 2008). Shade avoidance costs would also be without a benefit if all surrounding plants would be genetically similar and thus show the same growth responses. No selective advantage exists in this scenario. Such a scenario occurs in crop monocultures: the shade avoidance responses that the individuals show do not improve their competitive position since surrounding plants show the same response.

Shade avoidance responses in crops, even have a negative impact on crop performance because resource investments are rerouted from harvestable organs towards stem elongation (Boccalandro et al., 2003; Carriedo, Maloof, & Brady, 2016; Robson, McCormac, Irvine, & Smith, 1996). In addition to preserving energy, it has also been proposed that in monocultures, inhibition of shade avoidance responses might increase the competitive performance of the entire community against other plants invading the monoculture, such as weeds in cropping systems (Weiner, Andersen, Wille, Griepentrog, & Olsen, 2010). This idea is rooted in the proposition that group fitness (of the whole monoculture field) can be optimized by a different set of traits than individual plant fitness in mixed vegetations. The, so far theoretical, mechanism is simple: Shade avoidance responses, such as more erect leaves, stem elongation and reduced tillering would not only bring the leaf tips closer to the light, but would also create a more open vegetation structure that facilitates light penetration. Invading plants, such as weeds, will benefit from this extra light and grow more vigorously. It has thus been proposed that reducing shade avoidance in a monoculture vegetation stand would promote group fitness against invading competitors (Denison, 2012; Weiner et al., 2010).

This hypothesis has, however, never been tested experimentally. Here, we will combine experiments and computational modelling to investigate if modulation of shade avoidance responses at high planting density can improve performance of a monoculture community against invading competitors by enhancing the monoculture's shading capacity. We will use wild type and shade avoidance mutant plants of *Arabidopsis thaliana* to investigate if modulation of shade avoidance characteristics can optimize vegetation architecture to suppress competitors; an opportunity that does not yet exist in other plant species. Our data show that indeed, inhibition of a shade avoidance response in a *pif7* mutant monoculture enhances community performance, and leads to more effective suppression of invading competitors. We conclude that modifying vegetation architecture through altered shade avoidance characteristics provides a novel opportunity to improve competitive performance of a monoculture against invading competitors.

## 2 MATERIALS AND METHODS

### 2.1 Plots, growth and measurements

Genotypes used in this study, as dominant vegetation plants were wild‐type Col‐0, *pif4‐101 pif5‐1* (Lorrain et al., 2008) and *pif7* (Leivar et al., 2008), whereas *pif4‐101 pif5‐1 pif7‐1* (de Wit et al., 2015) triple mutant plants were used as the invading competitor. Canopy seeds were sown in a pot with a surface area of 10.5x10.5 cm filled with a substrate of soil: perlite (2:1), with additional nutrients [6 g of slow release fertilizer (Osmocote “plus mini” Ammonium Nitrate Based Fertilizer; UN2071; Scotts Europe BV, Heerlen, The Netherlands) and 6 g MgOCaO (17%; Vitasol BV, Stolwijk, The Netherlands]. The *pif4pif5pif7* plants were sown in a different pot 3 days after canopy plants for germination. Sowing was followed by stratification for 4 days (dark, 4°C). After stratification plants were moved to a short‐day growth chamber (9 hr/15 hr of light/dark period respectively; R:FR was 2.3 and PAR = 150 μmol m^−2^ s^−1^). When the core vegetation plots were 15 days old (seeds were sown directly in plots), competitor *pif4pif5pif7* seedlings (12 days old) were transplanted into the plot (Figure S1), representing an invading competitor, or for example a weed in crop fields. The vegetation plots were grown for another 29 days and subsequently harvested. Measurements were performed on five plots where four canopy and four competitor plants were harvested (20 canopy plants and 20 competitor plants in total) from the middle of the plot to avoid any edge effects (Keuskamp, Pollmann, Voesenek, Peeters, & Pierik, 2010; Pierik, Visser, de Kroon, & Voesenek, 2003; Pierik, Whitelam, Voesenek, De Kroon, & Visser, 2004; Schmitt et al., 1995). Petiole and lamina length of the three longest leaves from each plant were measured with a digital caliper. Individual plant leaf area (20 plants/genotype) was scanned and determined with image‐J software after carefully being harvested from the middle of the plots. Shoot dry weight was recorded with a digital scale, after drying the tissue at 70°C oven for 3 days. Plot biomass (g/m^2^) and Leaf Area Index (LAI) were calculated from the same four individuals per plot (five plots in total) by extrapolating to the full plot and density [average of dry weight from the harvested plants of each density * the plant density (plants/m^2^)]. The heights of the plots were measured with a ruler while an independent determination of the canopy cover was derived from top photographs using the PlantCV software (Gehan et al., 2017). In the canopy cover measurements by the PlantCV, we exclude the outer plants of the vegetation stand to avoid edge effects. This measurement shows in a vertical projection which percentage of space is being covered by the plants. The height and the canopy cover measurements were taken every 5 days, starting from day 20 of growth. Reproductive output was recorded in separate experiments with the same growth conditions, 3 months after sowing. Every 10 days (starting from the sowing day) plants were watered with nutrients, on all other days they were watered with tap water. When the first silique from each pot turned brown, watering was stopped. The number of siliques was measured, after 2 weeks of ripening. Differential petiole length of the fifth‐youngest leaf was recorded with the digital caliper for 13 days, starting at day 28 (day 0) and measurements from day 0 were subtracted from the measurements on the same individual leaf at any of the subsequent measurement days. Differential petiole angles (hyponasty) of the fifth‐youngest leaf were measured digitally with image J (according to Pantazopoulou et al., 2017). Since leaf angles in control plants are stable over the photoperiod and R:FR‐induced hyponasty is approximately at maximum in the morning (Figure S2), we took pictures every day at 10:00 a.m. (ZT = 2) for 13 days, starting at day 28 (*t* = 0). For each individual leaf, the angle at day 0 was subtracted from the recorded angle at day *x*, delivering the change in petiole angle. The relative differential petiole angle and length were calculated by subtracting the differential petiole angle or length of white light (control conditions) from the differential petiole angle or length of the different light treatments per timepoint and genotype. The fifth‐youngest leaf was selected in order to monitor the petiole angle and elongation changes through time since at this developmental stage the leaves are responsive to low R:FR and are sufficiently developed to allow reliable measurements, but have matured more than the still younger leaves. This standardization has been used in previous studies (Pantazopoulou et al., 2017).

### 2.2 R:FR measurements

The R:FR measurements started at day 20 (before the competition starts, (de Wit et al., 2012)] by using the Spectrosense2‐Skye light sensor with a glass fibre extension with 0.6 cm light collection area (R, λ = 655–665 nm; FR, λ = 725–735 nm). The sensor was placed inside the plot (Figure S1A) and measured the R:FR from four different directions and on four different positions, resulting in 16 measurements per time/per pot. When canopy closure occurred, the sensor was placed under the canopy, without causing any damage to the plants or interfering with the canopy light distribution. The measurements were always taken from the same position in all densities and patterns.

### 2.3 Experimental design of the densities and patterns with or without the competitor *pif4pif5pif7*

For the Col‐0 core vegetations three different densities were used (16 plants per pot (1,111 plants m^−2^), 25 plants per pot (2,500 plants m^−2^), 64 plants per pot (8,264 plants m^−2^); hereafter low, medium and high density respectively) and two spatial patterns [uniform (checkerboard design: equal distance between the plants) and row (bigger distance between the rows of the plants but smaller distance between the plants within the rows), See Figure S1B)]. In uniform pattern, the distance between the plants was 3, 2 and 1 cm in low, medium and high density respectively. In row pattern, the distance between the rows was always 5 cm while within the rows the distance between the plants were 0.6, 1.25 and 2 cm in high, medium and low density respectively. For the vegetations consisting of *pif4pif5 and pif7* plants only high density‐uniform pattern was used. The number of *pif4pif5pif7* competitor plants and spatial positions were the same in all the plots [16 plants per pot (1,111 plants m^−2^)] and they were transplanted into the high density‐uniform pattern plots 3 days later than the canopy plants (Figure S1C).

### 2.4 Light experiments

Individual plant responses to R:FR were studied for the different genotypes used here. To reduce the R:FR light ratios in the control white (W) light conditions (Philips HPI lamps [R:FR = 2.3, 160 μmol m^−2^ s^−1^ PAR]), supplemental far‐red LEDs (Philips Green Power FR 730 nm) were used. FR supplementation resulted in R:FR = 0.2 (160 μmol m^−2^ s^−1^ PAR). To mimic true canopy shade, green filter (Lee filters Fern Green) was used (resulting in R:FR = 0.35 and 35 μmol m^−2^ s^−1^ PAR). The light spectra of the treatments were measured with an Ocean optics JAZ spectroradiometer (Figure S3).

### 2.5 FSP model

A functional‐structural plant (FSP) model (Vos et al., 2009) of Arabidopsis rosettes, previously used and described in (Bongers et al., 2019; Bongers, Pierik, Anten, & Evers, 2018; Pantazopoulou et al., 2017), was used to simulate Arabidopsis plant types, using the simulation platform GroIMP and its radiation model (https://sourceforge.net/projects/groimp/). Arabidopsis rosettes were represented by a collection of leaves (represented by a petiole and lamina) whose appearance rate and shape were based on empirical data (Bongers et al., 2018). The leaves individually grew in time in 3D based on light interception, photosynthesis and plant‐wide carbon allocation principles (for detailed explanations of the principles seeBongers et al., 2018; Evers, 2016). In addition, leaves showed petiole elongation and hyponastic responses based on the virtual touching of leaves and the perception of R:FR (Bongers et al., 2018). Therefore, individual growth and shade avoidance responses depended on the capture of light (represented by PAR intensity) and the perception of R:FR within the simulated vegetation stand.

The light source emitted PAR representing 220 μmol m^−2^ s^−1^ and a R:FR ratio of 2.3, which corresponded to the growth chamber experiments. The simulated vegetation stands (representing the canopy plants) consisted of 100 plants that were placed in a uniform grid of 10 × 10 with an inter‐plant distance of 2.5 cm (1,600 plants m^−2^, similar as model calibration and validation, Bongers et al., 2018). In addition, if required in the model scenario, 16 competitor plants were placed between the canopy plants, similar as in the experiment (Figure S1C). Plants grew for 44 days based on the PAR captured, photosynthesis rates and carbon allocation patterns (Evers, 2016; Bongers et al., 2018). Each model time step, which represented 24 hours, hyponastic responses could occur if leaves touched or if the lamina tip was exposed to R:FR < 0.5 (Bongers et al., 2018; Pantazopoulou et al., 2017). The strength of the hyponastic responses differed per simulation run; plants could increase their leaf angle with 1, 5, 10, 15 or 20° per day. The angle of the leaves over time was therefore a function of the number of times in which touch and/or low R:FR perception occurred per individual leaf, with a maximum leaf angle of 80°.

In total three scenarios with each five different runs were simulated and 10 vegetation stands were simulated per scenario and run. *Scenario 1*) vegetation stands without competitor plants were simulated to quantify the PAR intensity at soil‐level during vegetation development. In scenario 2 and 3, vegetation stands that included competitor plants were simulated, in which the competitor plants had weak SAS (*Scenario 2*) or strong SAS (*Scenario 3*). Per scenario there were five different runs in which the vegetation plants differed in their ability to show hyponasty; plants increased their leaf angle with 1, 5, 10, 15 or 20° per day when sensing neighbours (with a maximum final leaf angle of 80°). Competitor plants with weak SAS could not show hyponastic responses (angle increase = 0), while competitor plants with strong SAS showed hyponasty with 16° per day (see “p4p5p7” and “Col‐0” simulated plant types in Bongers et al., 2018). Competitor plants with weak SAS showed slower petiole elongation than the core‐vegetation plants and competitor plants with strong SAS.

The intensity of PAR reaching the soil was captured by virtual soil‐tiles underneath the middle 16 core‐vegetation plants (representing an area of 100 cm^2^). Simulated above‐ground plant biomass (g/m^2^) was based on the middle 16 core‐vegetation plants and average competitor plant biomass (g) was based on the middle four competitor plants to avoid edge effects. Competitor plants germinated 3 days after the canopy plants, similar to the experiment.

### 2.6 Statistics

Data were analysed by one or two‐way ANOVA followed by LSD test. All the analyses were performed with GraphPad.

## 3 RESULTS

### 3.1 The effect of planting density and pattern on Col‐0 performance

**FIGURE 1:**
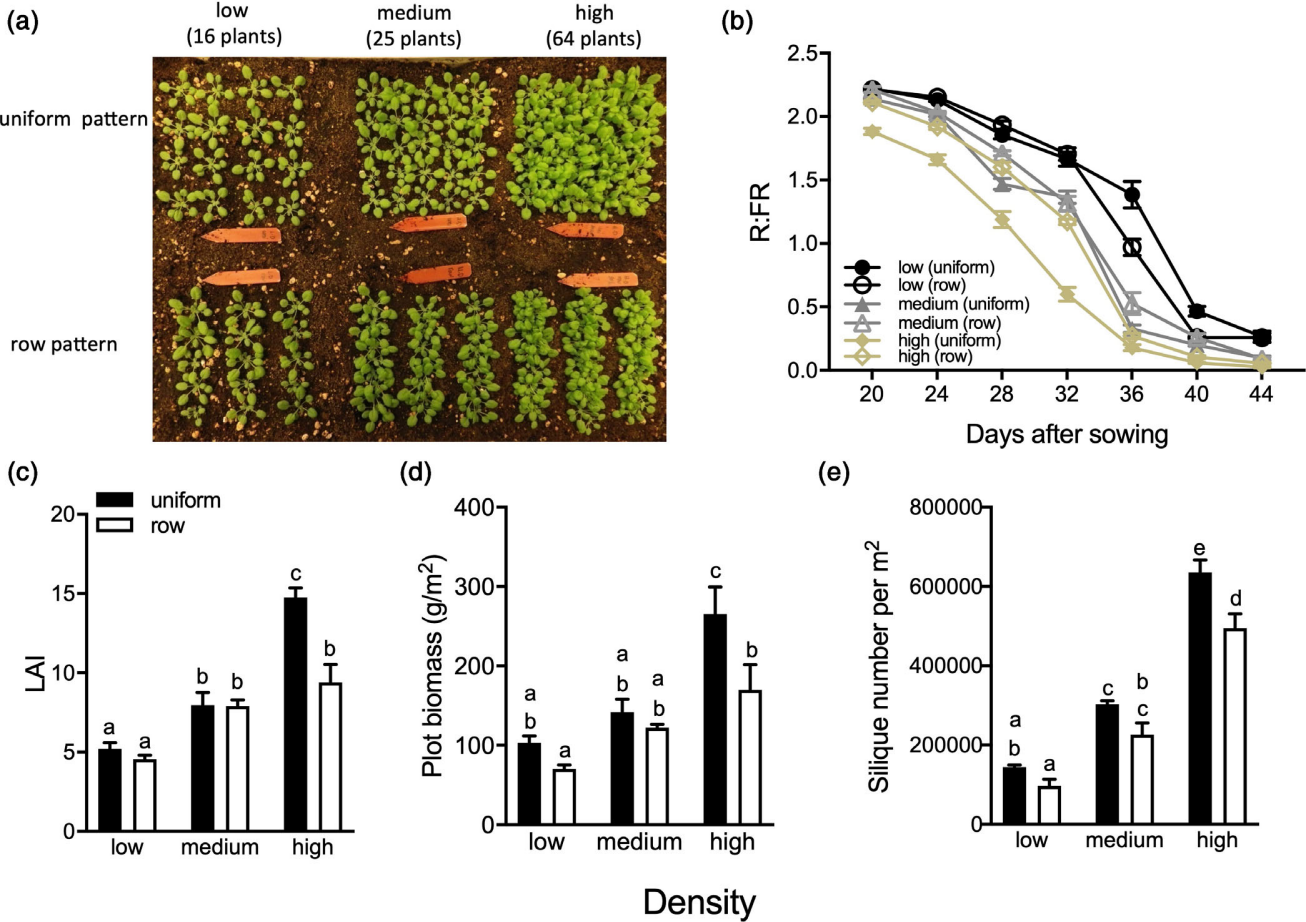
Arabidopsis Col‐0 in high density, uniform pattern produces more biomass and canopy cover than at lower densities and row patterns. (a) In the upper row Col‐0 plants grow in a uniform pattern (uniform), while in the lower row plants grow in row pattern (row) at three different densities (low, medium, high). (b) The R:FR light ratio measured inside Col‐0 canopies, during the days of growth, in low (black lines), medium (grey lines) and high (yellow lines) densities and two patterns (uniform and row). (c‐e) Leaf area index (LAI; c), plot biomass per m^2^ (d) and seed output (silique number per m^2^ pot; e) at three different densities (low, medium, high) and two different planting patterns (uniform, row). Data represent mean ± *SE* (*n* = 5). Different letters indicate statistically significant differences (two‐way ANOVA with LSD test, *p* < .05) [Colour figure can be viewed at wileyonlinelibrary.com]

To investigate the effect of sowing pattern and density on *Arabidopsis thaliana* (hereafter Col‐0) performance, we grew plots in three different densities (low, medium and high) and two different patterns (uniform and row) (Figure 1A). The R:FR showed a reduction in all densities and patterns through time, reflecting the growing canopy (Figure 1B). However, the strongest and most rapid decline of R:FR was observed in high density/uniform pattern, where the R:FR was decreased from approximately 2.0 to 1.1 after 8 days of measurements hinting at a rapidly closing canopy (Figure 1A). This was not the case for the row pattern in high density, where the R:FR was still high, presumably because the inter row distance was higher than in the uniform pattern. Low and medium density showed reduction of R:FR (less than 1.5) at day 36 (Figure 1B), indicating that the canopy remained more open for a longer period of time. The leaf area index (LAI) expresses the amount of leaf area per unit soil area and partially reflects the closure status of the canopy. We found that LAI increased more strongly at the high density/uniform pattern while high density/row pattern had similar LAI as medium density. LAI of the low density canopy plants was the smallest compared to the other densities in both planting patterns (Figure 1C). Interestingly, leaf lamina length decreased with increasing plant density, irrespective of the planting pattern (Figure S4A). The opposite was observed for petiole length, where the high density induced the strongest elongation (Figure S4B). Enhanced petiole elongation, combined with reduced lamina size, are classic aspects of shade avoidance.

Furthermore, there was a strong and significant effect of the density and planting pattern on Col‐0 biomass. The row pattern produced Col‐0 plants with smaller dry weight and leaf area (Figure S4C and S4D), most likely because the intraspecific competition was higher in rows compared to the uniform pattern since plants are much closer together within the rows. In terms of planting density, the total biomass of the plot in high density and uniform pattern was higher than the other densities (medium, low) than the row pattern (Figure 1D). Also, the number of siliques per square meter for the different density and planting patterns was consistent with biomass (Figure 1D and E). All together suggests that the uniform‐planting pattern at the high density would result in the highest reproductive output per unit area.

### 3.2 Canopy development and closure are affected by shade avoidance

**FIGURE 2:**
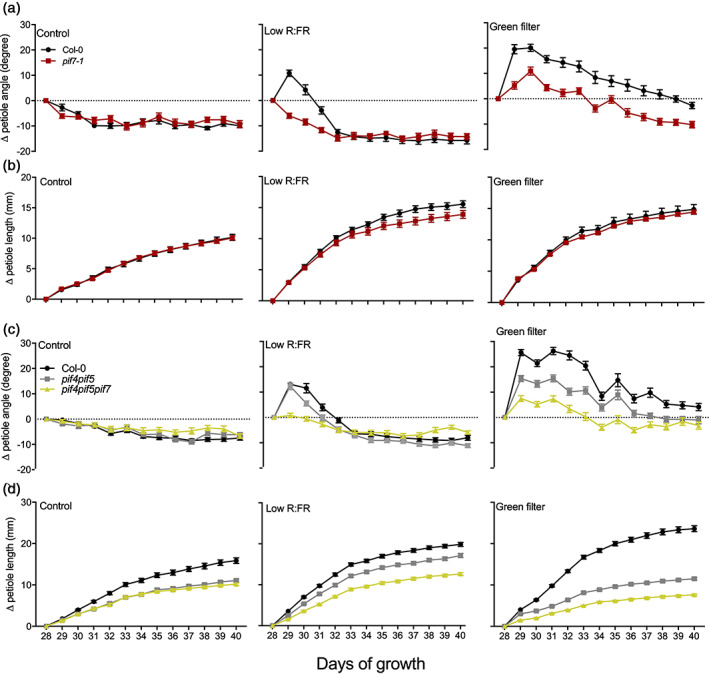
Shade avoidance responses (change in petiole angle (a, c) and change in petiole length (b, d)) of Col‐0, *pif7*, *pif4pif5* and *pif4pif5pif7* upon white light (control), low R:FR and green filter exposure. Light treatments lasted 13 days and started when plants were 28 days old. Data represent mean ± *SE* (*n* = 15). Differences in petiole angle and length responses between light treatment and light control per genotype and timepoint are shown in supplemental Figure S5 [Colour figure can be viewed at wileyonlinelibrary.com]

The high‐density uniform pattern clearly delivered the strongest Col‐0 canopy performance. We next investigated if alterations in hyponastic growth can regulate canopy closure and light penetration. To do that, we selected previously published mutants with altered shade avoidance characteristics. The *pif7*, *pif4pif5* double and *pif4pif5pif7* triple knockouts have reduced hyponastic responses to shade cues in short‐term experiments (Pantazopoulou et al., 2017) and we verified their responses to prolonged shade cue conditions. Reduction in R:FR resulted in the elevation of Col‐0 petiole angle (hyponasty) during the first 2 days (day 29 and 30) after which the response faded out, while petiole elongation was promoted from day 28 until 34 (Figure 2A‐D, and Figure S5 for relative differences). This fading out of the amplitude of FR‐induced hyponasty is consistent with observations on long‐day grown Arabidopsis in Michaud et al., 2017. Previously we have shown that within 24 hr of locally sensed low R:FR there is elongation of the abaxial side of the petiole that leads to hyponasty (Pantazopoulou et al., 2017). It is possible that in conditions of long‐term, whole‐plant low R:FR‐exposure, petiole elongation occurs on both sides (abaxial and adaxial), reducing the potential for hyponasty that results from petiole elongation on just the abaxial side. Green filter, mimicking real canopy shade, on the other hand elicits a continuous combination of hyponasty and petiole elongation in Col‐0 from day 28 up to 36 (8 days) (Figure 2B and D). *pif7* had a similar petiole elongation response as did Col‐0 in all the treatments but its hyponastic response to low R:FR (cue for early neighbour detection) was entirely absent, whereas its response to green shade (reproducing canopy shade) was severely reduced (Figure 2A and B, Figure S5A and S5B). *pif4pif5* showed a phenotype initially similar to wild type both in terms of petiole angle and elongation, but the petiole elongated clearly less in response to green filter treatment (Figure 2C and D and Figure S5C and S5D). On the other hand, *pif4pif5pif7* was unresponsive to low R:FR for both traits (Figure 2C and D and Figure S5C and S5D). Hyponastic responses were reduced in *pif4pif5* and not observed at all in *pif7 and pif4pif5pif7* under these severe shade conditions (Figure 2A and C and Figure S5A and S5C). We also verified growth of Col‐0, *pif7*, *pif4pif5* and *pif4pif5pif7* under white light and found that *pif7* had dry weight and leaf area that were not significantly different from Col‐0 wild type. *pif4pif5* had reduced leaf area but the dry weight was similar to Col‐0 even though there seems to be a trend towards reduced growth in the *pif4 pif5* double mutant compared to wild‐type Col‐0. On the other hand, *pif4pif5pif7* showed reduced growth compared to Col‐0 (Figure S6). In summary, Col‐0 shade avoidance responses (hyponasty and petiole elongation) were stronger in green shade than in low R:FR alone. Overall, *pif4pif5* was less responsive than Col‐0, whereas *pif4pif5pif7* was fully insensitive to the different light conditions. Interestingly, *pif7* showed similar petiole growth as Col‐0 and a similarly absent hyponastic response as in *pif4pif5pif7*.

**FIGURE 3:**
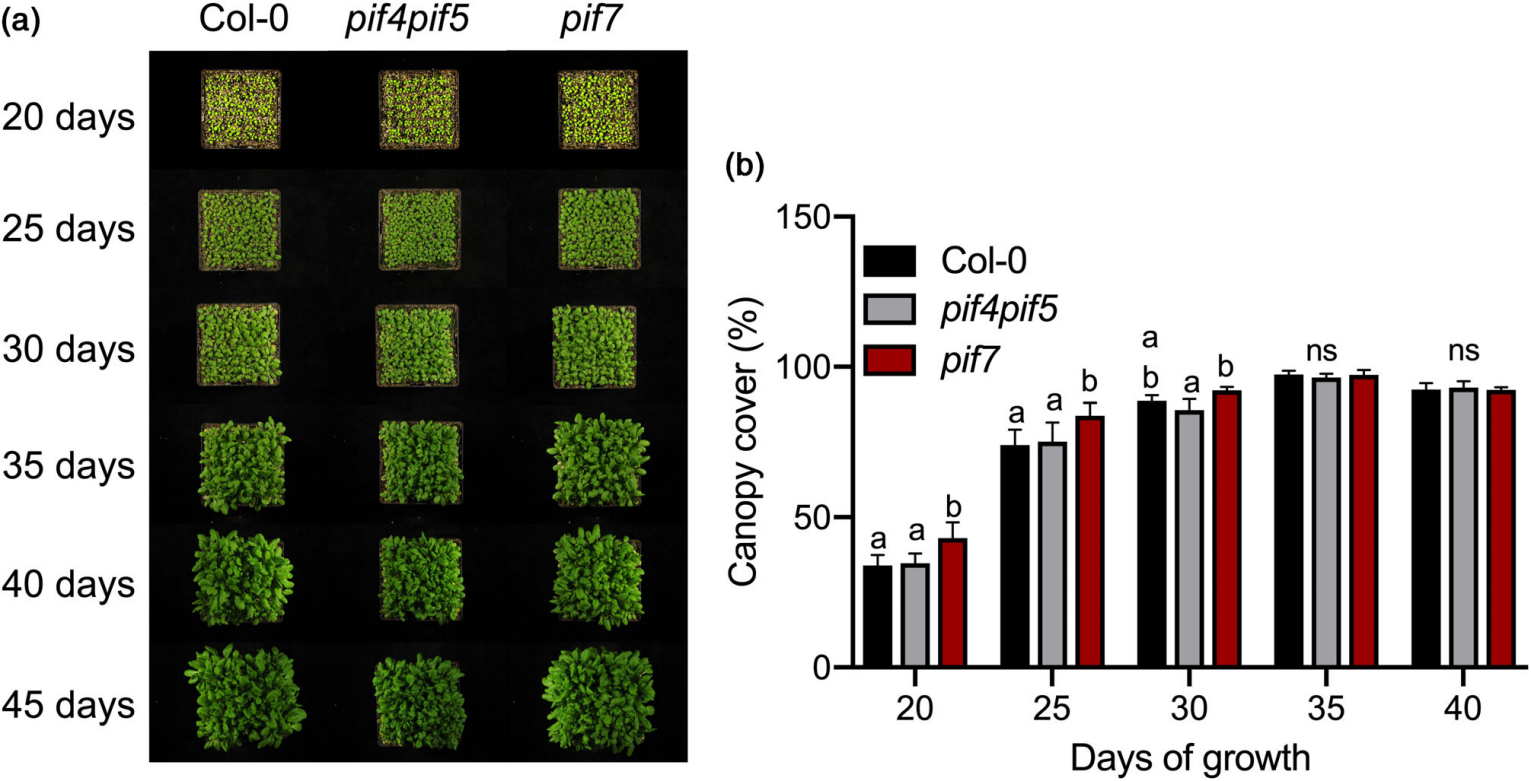
The *pif7* mutant creates a faster closed canopy than Col‐0 and *pif4pif5*. (a) Pictures illustrate how the canopies of Col‐0 (left), *pif4pif5* (middle) and *pif7* (right) plants develop and close soil exposure to light. (b) The percentage of soil covered by the same canopies: Col‐0 (black bars), *pif4pif5* (grey bars) and *pif7* (red bars) plants, through time. The Col‐0, *pif4pif5* and *pif7* canopies plants grew at high density, uniform pattern. Data represent mean ± *SE* (*n* = 5). Different letters indicate statistically significant differences (two‐way ANOVA with LSD test, *p* < .05. ns = not significant) [Colour figure can be viewed at wileyonlinelibrary.com]

The impact of different magnitudes of hyponastic responses in canopy closure, was tested by growing canopies of Col‐0, *pif7* and *pif4pif5*. High density, uniform planting patterns were used, since these closed their canopies most effectively (Figure 1). We monitored the canopy closure state through time by using the imaging analysis software tool PlantCV (Figure 3). We found that *pif7* canopies developed a better soil cover than Col‐0 and *pif4pif5* early in canopy development (day 20 until 25). The *pif4pif5* canopies remained more open than *pif7* canopies for another 5 days but percentage of the covered soil area was not significantly different from Col‐0 canopies at day 30. At later stages all canopies had developed nearly full closure. The *pif4pif5* canopies display reduced petiole elongation compared to *pif7* and Col‐0 (Figure S7), resulting in a relatively low canopy height for this double mutant (Figure S8). The height of *pif7* canopies was also reduced as compared to Col‐0 (Figure S8), presumably because of the reduced upward leaf movement in this mutant (Figure 2A and Figure S5A)

### 3.3 Performance of canopy and competitor plants during competition

**FIGURE 4:**
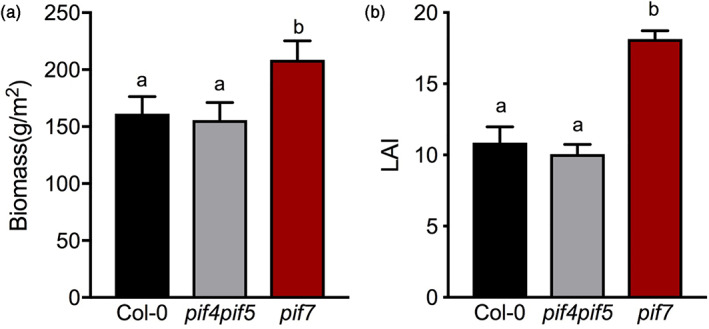
The *pif7* canopies grew larger than Col‐0 and *pif4pif5* under high‐density competing conditions. (a) Biomass and (b) LAI of canopies consisting of Col‐0 (black bar), *pif4pif5* (grey bars) or *pif7* (red bars), growing at high density, uniform pattern, measured after 44 days of growth. Data represent mean ± *SE* (*n* = 5). Different letters indicate statistically significant differences (two‐way ANOVA with LSD test, *p* < .05) [Colour figure can be viewed at wileyonlinelibrary.com]

**FIGURE 5:**
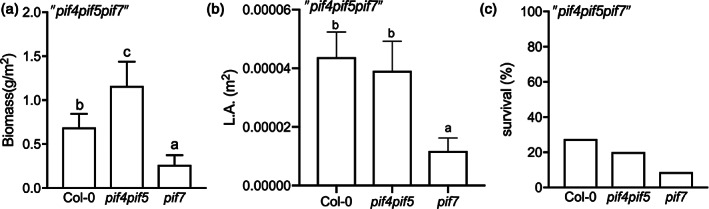
The *pif7* canopies suppressed the competitor*pif4pif5pif7* more effectively than did Col‐0 and *pif4pif5* canopies. The competitor's (a) biomass, (b) leaf area and (c) percentage of survival, under the canopies of Col‐0, *pif4pif5* and *pif7* for 44 days. The plants grew at high density, uniform pattern. Data represent mean ± *SE* (*n* = 5). Different letters indicate statistically significant differences (two‐way ANOVA with LSD test, *p* < .05) [Colour figure can be viewed at wileyonlinelibrary.com]

To test the impact of separate shade avoidance traits on competitor suppression and canopy performance, we grew different canopies: Col‐0, *pif4pif5*, and *pif7*. As an invading competitor we used *pif4pif5pif7*, which was planted between the canopy plants. *pif4pif5pif7* is unresponsive to shade (Figure 2C and D), ensuring that it will remain under the canopies and can be used to test the effect of different canopy architectures on competitor performance. To estimate shade avoidance responses of the different genotypes in true canopies, rather than independent light treatments, we measured petiole and lamina length at the end of the canopy development. *pif7* canopy plants displayed the largest lamina compared to Col‐0 and *pif4pif5* canopy plants during competition. Petiole length was enhanced upon competition in Col‐0 and *pif7* but not in *pif4pif5* canopy plants (Figure S7A and S7B). The strong lamina and petiole elongation but not hyponastic response of *pif7* during competition could have resulted in the higher biomass and LAI derived from the higher individual dry weight and leaf area compared to the other two genotypes (Figure 4 and Figure S7C and S7D). This also had a strong effect on *pif4pif5pif7* competitor performance. The faster closed canopy and plant growth of *pif7* during competition was associated with a reduction in growth of *pif4pif5pif7* competitors (Figure 5A and B). On the other hand, the improved light exposure of *pif4pif5pif7* competitor plants under the not so rapidly closed canopy of *pif4pif5* was associated with enhanced biomass and leaf area of the competitor triple mutant compared to the other genotypes (Figure 3, Figure S5 and Figure 5A and B). Indeed, the *pif4pif5pif7* competitor hardly survives under the *pif7* canopy while the percentage of survival between Col‐0 and *pif4pif5* was similar (Figure 5C).

### 3.4 Reducing shade‐induced hyponasty improves canopy performance against competitors

**FIGURE 6:**
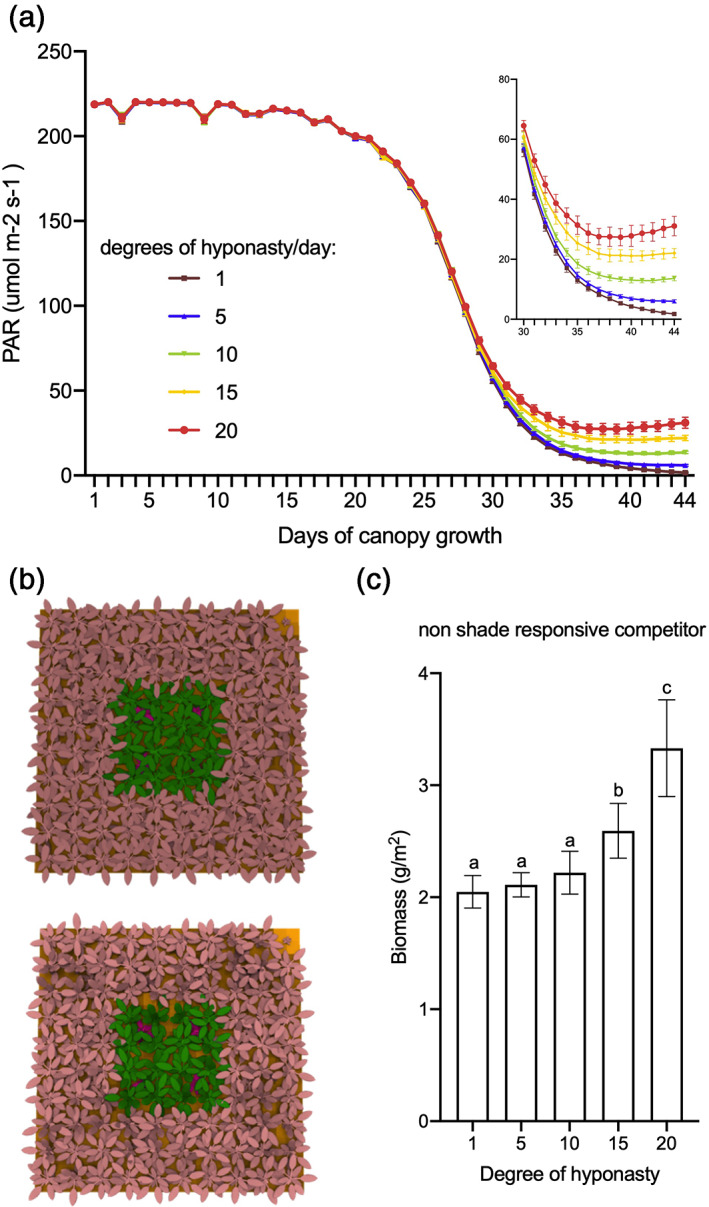
Reduced hyponastic responses result in lower light penetration and reduced competitor biomass. (a) Photosynthetically Active Radiation (PAR) of five different hyponasty scenarios is simulated with a 3D Arabidopsis computational model. Various canopy growth simulation scenarios consist of plants with different degrees of hyponastic responses to proximate neighbour plants, ranging from 1 to 20° per day. (b) Images illustrate the canopy cover over competitors, with a 1° hyponasty per day canopy (top) and a 20° hyponasty per day canopy (bottom) at day 30 of the simulations. The centre plants were used for quantifications, the surrounding rows in pink serve as edge plants. Canopy plants are depicted in green, invading competitors inside the green canopy are identified in red. (c) Simulated above‐ground biomass of the invading competitors grown in canopies with 1, 5, 10, 15 and 20° per day of hyponasty in response to neighbour detection. Biomass quantification was registered after 44 days. Data represent mean ± *SD* (*n* = 10). Different letters indicate statistically significant differences (one‐way ANOVA with LSD test, *p* < .05) [Colour figure can be viewed at wileyonlinelibrary.com]

Using a previously published 3D computational Arabidopsis canopy model (Bongers et al., 2018), we determined the light quantity [Photosynthetically Active Radiation (PAR)] in the canopy vegetation through time. As a feature of the canopy plants five different hyponastic scenarios were simulated; from 1° up to 20° hyponastic growth per day in response to neighbours (Figure 6A). The simulations show that canopies, consisting of plants with minimal hyponastic response to neighbours (e.g., 1, 5 and 10°) create strong reduction of light penetration inside the canopy; with PAR being reduced from 220 μmol m^−2^ s^−1^ to less than 60 μmol m^−2^ s^−1^ within 30 days, minimizing the amount of light reaching the soil. On the other hand, scenarios with faster hyponasty (e.g., 15 and 20°) allowed for less light extinction and thus higher PAR inside the canopy was observed. These simulations support the notion that upward leaf movement responses to neighbours may facilitate light penetration through the canopy, which can be beneficial for growth of invading competitors.

To test the effect of different magnitudes of hyponasty on the competitor's performance, we simulated these canopies again, but now with invading competitors. The competitors we used were unresponsive to shade (no hyponasty and reduced petiole elongation) to ensure that they will remain under the canopy. At the 15 and 20° per day hyponastic responses, the biomass of the competitor was increased while the biomass of the canopy was reduced compared to the hyponasty scenarios of 1, 5 and 10° per day (Figure 6B and C, Figure S9).

Based on these findings, reduced hyponastic responses can cause reduction of light penetration leading to suppression of the competitor and a potential increase of canopies biomass. The success of this communal repression of invading competitors would likely be contingent upon the invading competitor's ability to escape from the heavy shade cast by the dominant canopy. Indeed, when we introduced a shade avoiding competitor, rather than the non‐shade avoiding one used above, our simulations show that it benefits from lack of hyponasty in the dominant canopy, rather than being further suppressed by it (Figure S9). Overall, we conclude that a reduction of shade‐induced hyponasty of a dominant monoculture can help suppress competitors through enhanced shading capacity. However, if competitors are able to escape the dominant canopy through effective shade avoidance responses, this advantage is lost.

## 4 DISCUSSION

Crop sowing uniformity in high density can positively affect yield and suppress weeds as compared to a scenario where crops, for example wheat, are grown in rows (Kristensen, Olsen, & Weiner, 2008; Olsen, Kristensen, & Weiner, 2006; Weiner, Griepentrog, & Kristensen, 2001). We confirm this positive effect of a uniform pattern in high density‐grown Arabidopsis, as indicated by increased biomass and leaf area in uniform compared to row‐grown plants (Figure 1, Figure S4C and S4D). Indeed, when grown at a high density in rows, the plant–plant distance within the row is very small, leading to severe intraspecific competition within the rows. When a same density of plants is grown in a uniform planting pattern, the plant–plant distance is larger, leading to less severe interactions and increased, homogeneous performance (Weiner et al., 2001, 2010). Furthermore, the uniform pattern allows for a homogeneous closure of the canopy, leaving only very few spots where light reaches the soil and allow an invader to grow. In a canopy with row pattern, on the other hand, it takes a longer time for the canopy to close the large space between rows, allowing invading competitors to grow from the sunlight reaching the soil between the rows. We, therefore, expect that canopy shade avoidance manipulation, such as here with *pif* mutants, is particularly effective in a high‐density, uniform planting pattern. We suggest that this might indeed work in crops when grown in high density, uniform planting patterns as proposed by, for example, (Weiner et al., 2010). In addition to the proposed improved weed suppression, reduced shade avoidance investments would also allow more resource investments into harvestable organs (Carriedo et al., 2016). Despite the urgent need for crop improvement, well‐defined shade avoidance mutants have hardly been described in crops (Carriedo et al., 2016; Kebrom & Mullet, 2016; Weiner, Du, Zhang, Qin, & Li, 2017). Here, we tested the impact of shade avoidance modulation on canopy performance and weed suppression in the model species *Arabidopsis thaliana*. We show that indeed a canopy of plants with reduced shade avoidance properties has improved abilities to communally suppress invading competitors.

Although petiole elongation, combined with upward leaf movement (hyponasty), will increase access to light at the individual plant level (Ballaré & Pierik, 2017; Pantazopoulou et al., 2017), the reduced leaf lamina growth that typically occurs in shade avoiding *Arabidopsis* (de Wit et al., 2015) may counterbalance the potential gain in photosynthesis of individual plants (Fritz, Rosa, & Sicard, 2018). Part of the shade avoidance responses will have been triggered through the drop in R:FR inside the canopies (Figure 1B). However, shade avoidance responses, and especially hyponasty, can on their turn also affect the R:FR ratio, as well as other aspects of the light composition and availability, inside the canopy by affecting the extent to which a vertical canopy structure is formed in this otherwise horizontally growing rosette species (de Wit et al., 2012). Modulating shade avoidance traits in different canopy structures may thus affect light distribution inside these canopies. Indeed, using a 3D Arabidopsis plant model (Bongers et al., 2018), we found that slow‐down of hyponastic growth upon shade detection in a canopy monoculture can clearly reduce light penetration through the canopy down to soil level (Figure 6A). The 3D model also predicted that canopy plants with severely reduced hyponastic growth could suppress non‐shade responsive competitors while the biomass of the canopy plants can be increased (Figure 6C and Figure S9B). Consistently, our growth chamber experiments with wild type and *pif* mutants confirmed that reduced hyponastic response in the dominant canopy, can suppress growth of invading competitors (Figure 5A and C). The non‐hyponastic *pif7* canopy, in addition, also grew larger than a Col‐0 canopy (Figure 4A), which may be associated with the reduced investments in shade avoidance, and reduced competitive loss of resources to competitors. The observation that the size of *pif7* was not different from Col‐0 when plants were grown individually in pots (Figure S6) indicates that indeed this effect is caused by growth at high density and does not represent an overall growth rate difference between the genotypes. We propose that the faster closing of the *pif7* canopy, due to lack of low R:FR‐induced hyponasty, together with the larger LAI as compared to the Col‐0 and *pif4pif5* canopies (Figure 2, Figure S5A and S5B, Figure 3 and 4), resulted in less light availability for the competitor, leading to reduced performance of the competitor (Figure 5).

Interestingly, despite the fact that the *pif4pif5* canopy architecture showed mild reduction of shade avoidance responses, the competitor *pif4pif5pif7* performed similar in Col‐0 and *pif4pif5* canopy (Figure 5). We speculate that the advantage of modestly reduced shade avoidance in *pif4pif5* for communal competitor suppression might be outweighed by its reduced overall growth rate (Figure 2, Figure S5D), which still leads to a relatively open canopy (Figure 3). Future experiments on mutants with even more subtle variations in hyponasty, petiole elongation and overall growth rate would allow testing this explanation.

As mentioned above, the *pif4pif5pif7* triple mutant lacks any shade avoidance response to (combinations of) light signals that indicate plant density (Figure 2, Figure S5C and Figure SD). This allowed us to study if the dominant canopy architecture can be optimized such that growth in the understory can be inhibited by shading the invading competitors. Using the 3D model, we also verified performance of competitors that can show shade avoidance responses and thus have the capacity to compete stronger against the dominant canopy. The model showed that invading competitors that are of similar size as the dominant canopy and that can escape from the shade‐casting canopy altogether, may improve their growth at the expense of the collective performance of the dominant canopy (Figure S9). However moderate reduction of hyponasty (from 20° to 10°) caused a further reduction of competitor biomass while the opposite was observed for the canopy biomass (Figure S9). The reasons may be that in the slightly reduced hyponasty scenario, the canopy plants reduced the light penetration through the canopy and improved its own light interception due to a better leaf display angle relative to the incoming light.

If suitable mutants or transgenic lines come available for upright‐growing, stem‐forming plants, these could be used to test these scenarios experimentally, of a more vertically layered canopy, representing many of the staple crops world‐wide, for weed‐suppression.

To our knowledge, this study is the first to show that losing one of the shade avoidance responses in Arabidopsis canopies, hyponasty, has potential to suppress competitors. This is proof of concept of a major prediction in Evolutionary Agroecology (Weiner et al., 2010) and Darwinian Agriculture (Denison, 2012), stating that communal performance of a dominant canopy can be optimized by selecting against individual fitness and in favour of group performance. Translating our findings to crop‐weed competition scenarios may depend on the architecture of the crop and would require follow‐up studies that include mutants with reduced shade avoidance responses in upward‐growing, stem forming species. Such translation would also require subsequent testing of the ability of weeds to display shade avoidance responses and potentially outgrow the crops in scenario's where for example height growth of crop plants would be reduced. We, therefore, conclude to state that this proof of concept study on communal suppression of competitors by a canopy with reduced shade avoidance properties is promising, but its application in agriculture might depend on the precise architecture and shade avoidance properties of crops and weeds of interest, planting density as well as planting pattern, and is therefore likely to work in some, but not all cropping scenarios.

## CONFLICT OF INTEREST

The authors declare no conflict of interest.

## AUTHOR CONTRIBUTIONS

Chrysoula K. Pantazopoulou and Ronald Pierik designed research; Chrysoula K. Pantazopoulou and Franca J. Bongers performed research; Chrysoula K. Pantazopoulou and Franca J. Bongers analysed data; and Chrysoula K. Pantazopoulou and Ronald Pierik wrote the paper.

## Supporting information


**Appendix S1.** Supporting Information.
