# Supplementary material for: Reducing shade avoidance can improve Arabidopsis canopy performance against competitors
Source: Plant Cell Environ. 2020 Oct 19;44(4):1130–41. doi: 10.1111/pce.13905 (PMC8048483; doi:10.1111/pce.13905)
Supplement: Supplementary file 1 — Appendix S1. Supporting Information. [file PCE-44-1130-s001.pdf]

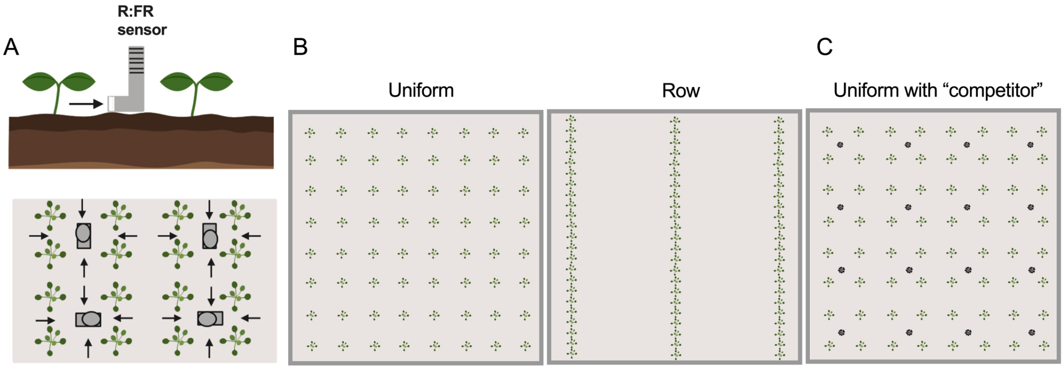

Figure S1: schematic overview of the method of R:FR measurements and sowing pattern. (A) A glass fiber R:FR sensor collected light inside the canopies, black arrows represent the direction of the measurements of each position. The sensor was placed in the four positions indicated in the bottom picture. (B) Schematic illustration of high sowing density at uniform and row sowing pattern. (C) Schematic illustration of the placement of the invading competitors (*pif4pif5pif7*, depicted in black) at high density - uniform pattern (total 16 competitors placed per pot). This figure was created with Biorender.

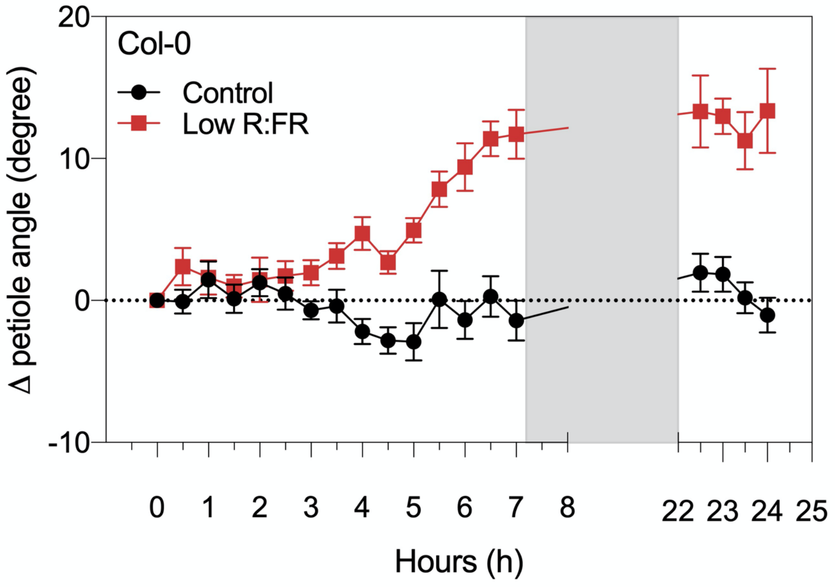

Figure S2: The petiole angle of Col-0 through time upon Low R:FR. Time-lapse of differential petiole angle of Col-0 in different light treatments [white light (control) and FR-enriched (Low R:FR)]. Grey bar represents the night period. Data represent mean ± SE (n=10).

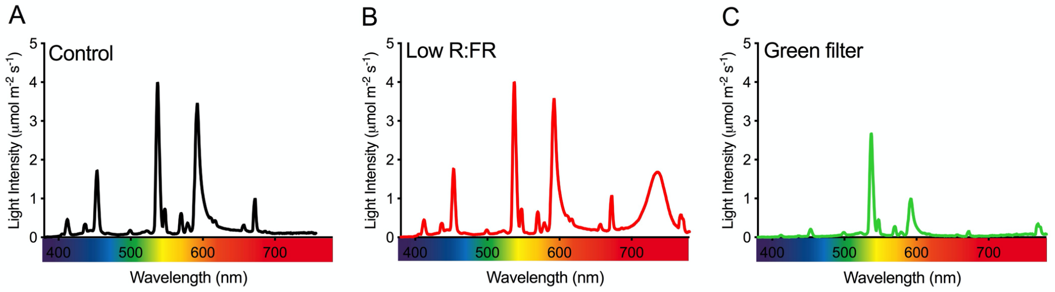

Figure S3: The spectral composition of the tree different light treatments: white light control (W), FR-enriched (Low R:FR) and green shade (Green filter) in the growth room.

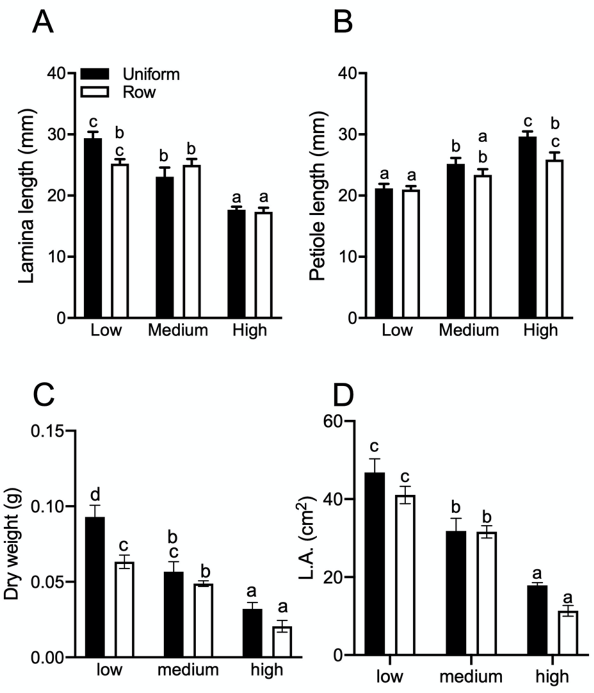

Figure S4: The effect of different densities on lamina and petiole length, dry weight and leaf area of Col-0. Leaf lamina (A) and petiole (B) length (C) dry weight and (D) leaf area of Col-0 plants in three different densities and two different sowing patterns after 44 days. Data represent mean ± SE (n=5). Different letters indicate statistically significant differences (two-way ANOVA with LSD test, $P < 0.05$).

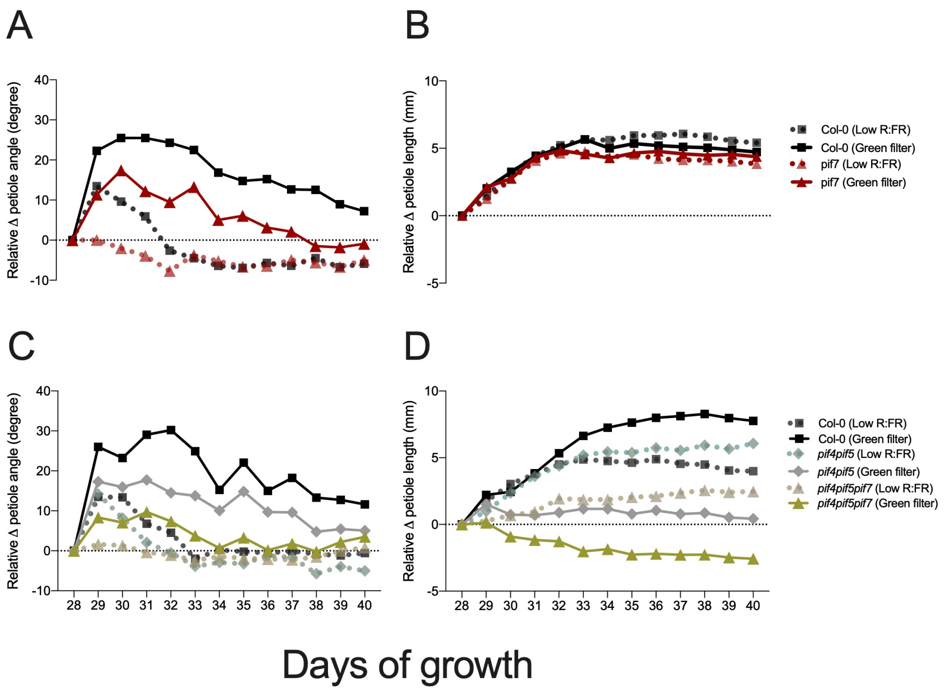

Fig. S5: The relative shade avoidance responses of Col-0, *pif7*, *pif4pif5* and *pif4pif5pif7* upon low R:FR and green filter exposure. A & C show the relative differential petiole angle and B & D the relative differential petiole length Light treatments lasted 13 days and started when plants were 28 days old. Values were calculated from data in Figure 2 by subtracting the average differential petiole angle (A, C) or length (B, D) of white light (control conditions) from the average differential petiole angle or length of the different light treatments per timepoint and genotype.

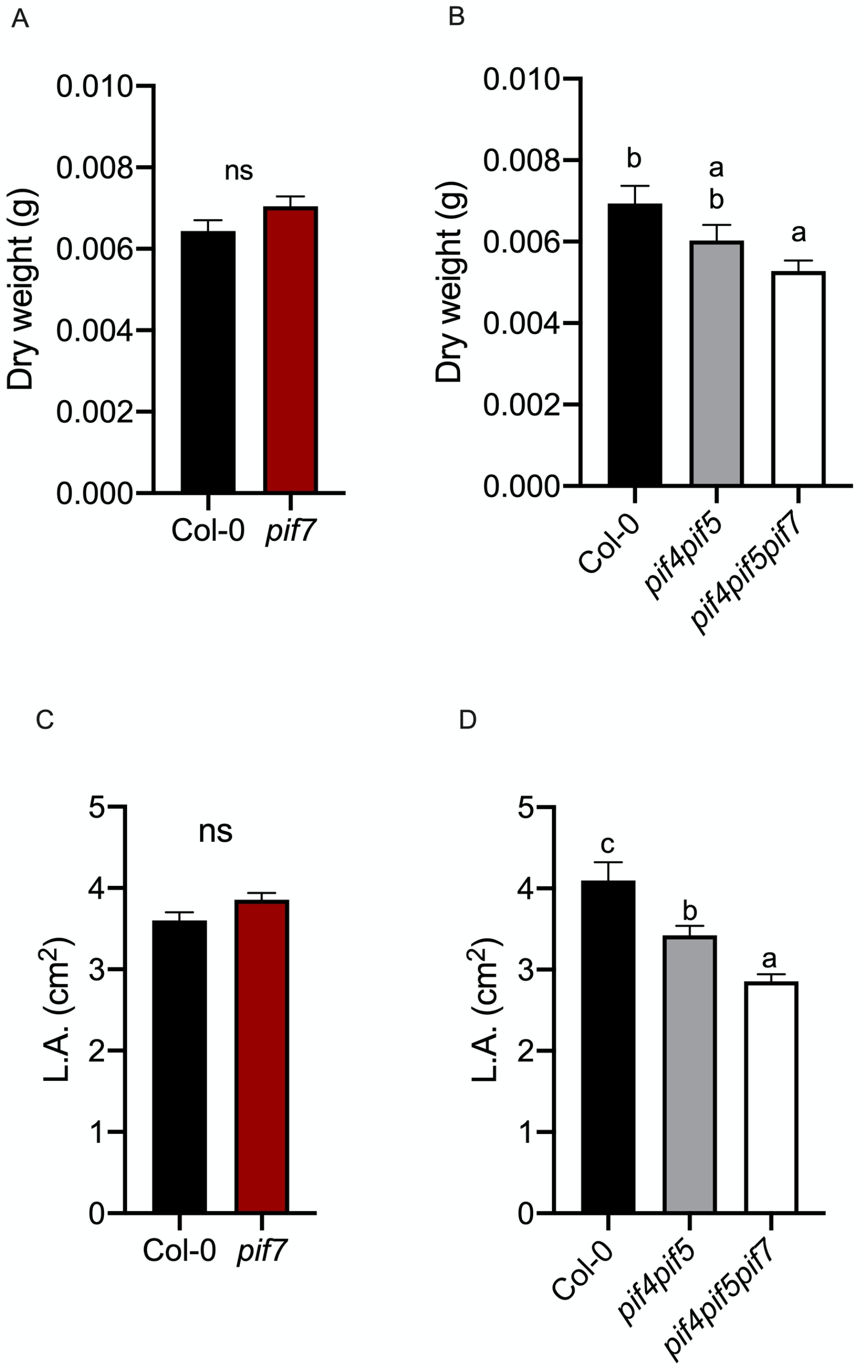

Figure S6: The Leaf area (L.A) and dry weight of Col-0, *pif7*, *pif4pif5* and *pif4pif5pif7* after growing for 28 days in white light. Dry weight (A, B) and leaf area (C, D) of *pif7* and *pif4pif5pif7* individuals is similar to Col-0. Plants were grown for 28 days under white light. Data represent mean ± SE (n = 8-10). Different letters indicate statistically significant differences (two-way ANOVA with LSD test, $P < 0.05$).

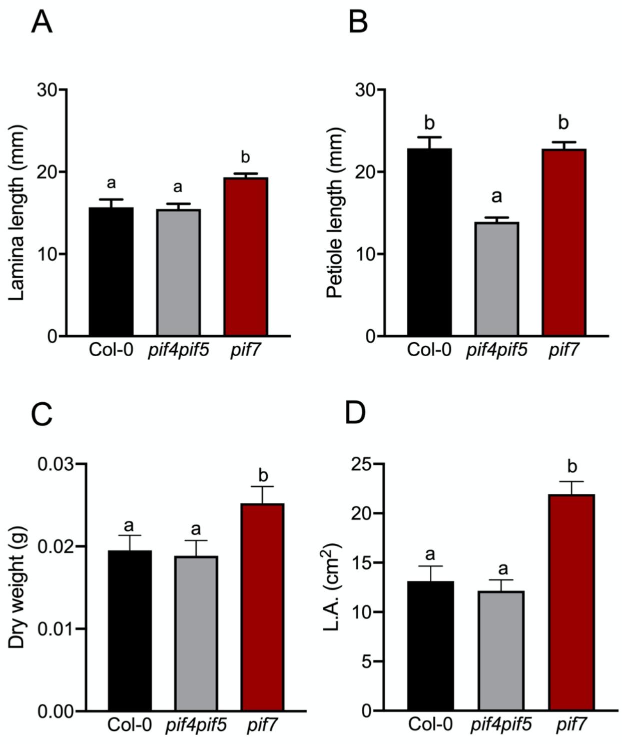

Figure S7: Effect of high density on Col-0, pif4pif5 and pif7 performance. Leaf lamina (A) and petiole (B) lengths, (C) dry weight and (D) leaf area of Col-0, *pif4pif5*, *pif7* growing in high density, uniform pattern with *pif4pif5pif7* competitors for 44 days. Data represent mean ± SE (n=5). Different letters indicate statistically significant differences (two-way ANOVA with LSD test, $P < 0.05$).

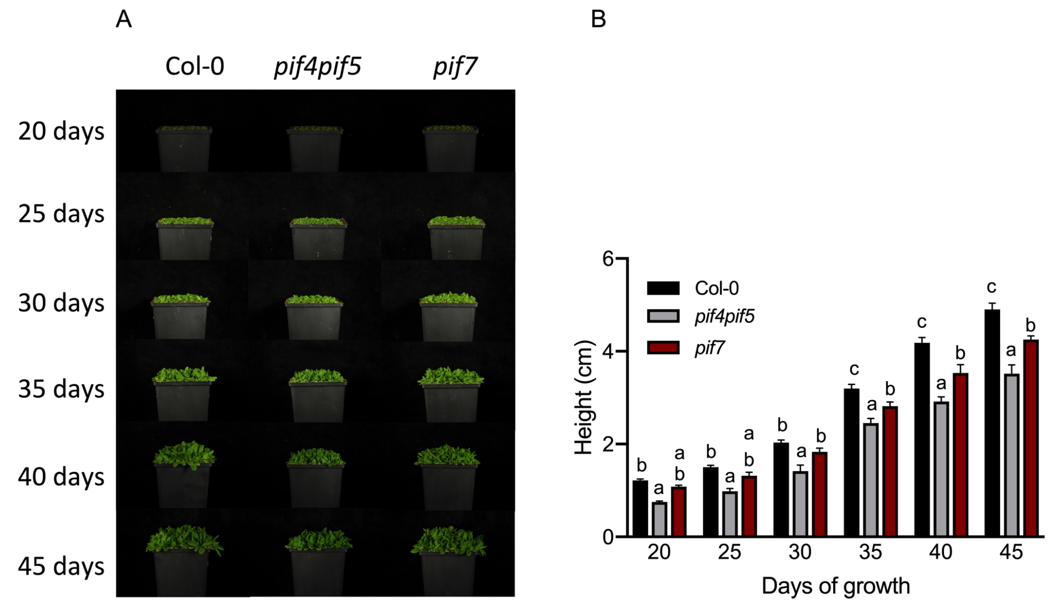

Figure S8: Col-0 canopies have higher canopy height than *pif4pif5 and pif7* canopies. (A) Pictures illustrate the canopies height of Col-0 (left), *pif4pif5* (middle) and *pif7* (right) through the time. (B) Canopy height of Col-0 (black bars), *pif4pif5* (grey bars), *pif7* (Red bars) canopies through time. These canopy plants are growing in high density, uniform pattern. Data represent mean ± SE (n=5). Different letters indicate statistically significant differences (two-way ANOVA with LSD test, $P < 0.05$).

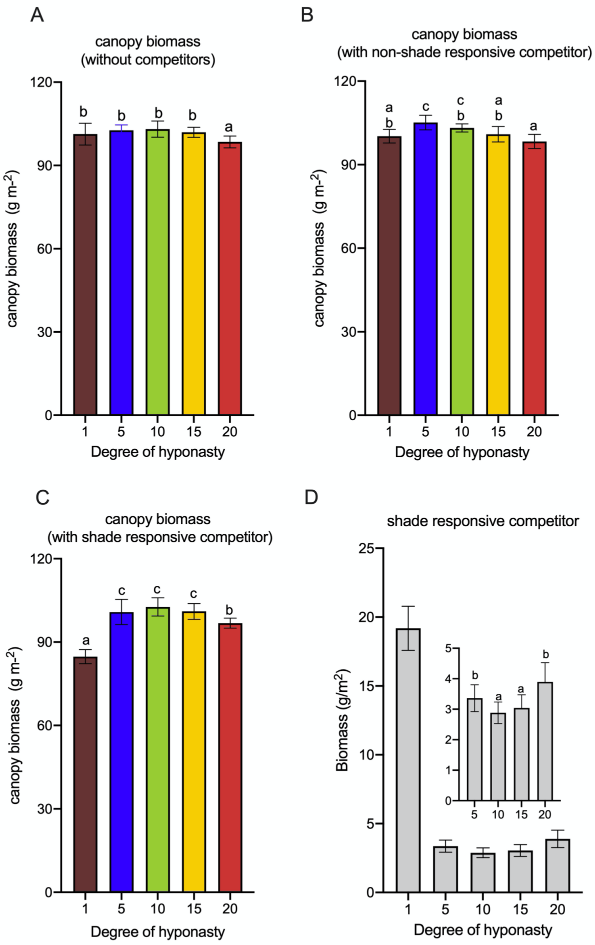

Figure S9: Canopy biomass can be affected by canopy leaf hyponasty. Canopy biomass was calculated in a 3D computational model with five hyponasty scenarios (1,5,10,15 and 20 degrees) in canopies without competitor (A), with non-shade avoiding competitor (B) and with shade avoiding competitor (C) after 44 days of canopy growth. (D) depicts shade responsive competitor biomass under canopies with 1,5,10,15 and 20 degrees of hyponasty after 44 days of canopy growth. Data represent mean ± SD (n=10). Different letters indicate statistically significant differences (one-way ANOVA with LSD test, $P < 0.05$).
